# Frugal filtering optical lenses for point-of-care diagnostics

**DOI:** 10.1364/BOE.381014

**Published:** 2020-03-09

**Authors:** Joanna Long, Helen E. Parker, Katjana Ehrlich, Michael G. Tanner, Kevin Dhaliwal, Bethany Mills

**Affiliations:** 1 EPSRC Proteus IRC Hub in Optical Molecular Sensing and Imaging, Centre for Inflammation Research, Queen's Medical Research Institute, University of Edinburgh, Edinburgh, EH16 4TJ, UK; 2 Department of Applied Physics, Royal Institute of Technology, Stockholm, Sweden; 3 Scottish Universities Physics Alliance (SUPA), Institute of Photonics and Quantum Science (IPAQS), Heriot-Watt University, Edinburgh, EH14 4AS, UK

## Abstract

Infectious diseases are the leading cause of morbidity and mortality in low and middle income countries (LMICs). Rapid diagnosis of infections in LMICs presents many challenges, especially in rural areas where access to health care, including diagnostics, is poor. Microscopy is one of the most commonly used platforms to diagnose bacterial infections on clinical samples. Fluorescence microscopy has high sensitivity and specificity but to date is mostly performed within a laboratory setting due to the high-cost, low portability and highly specialist nature of equipment. Point-of-care diagnostics could offer a solution to the challenge of infection diagnosis in LMICs. In this paper we present frugal, easy to manufacture, doped polydimethylsiloxane filtering optical lenses that can be integrated into smartphone microscopes for immediate detection of fluorescently labelled bacteria. This provides a breakthrough technology platform for point-of-care diagnostics.

## 1. Introduction

Sepsis and other bacterial infections are a leading cause of mortality globally, with the greatest burden affecting low and middle income countries (LMICs) [1]. Point-of-care (POC) diagnostics will enable faster decision making and timely treatments [2]. However, diagnosing infections in low resource settings presents many challenges which include technological and infrastructural limitations alongside integration into care pathways [1–3].

In the absence of bacteriological confirmation many infections are treated empirically, which can add to the global issue of antimicrobial resistance [4]. Some diagnoses, such as the initial screening for suspected *Mycobacterium tuberculosis* (Mtb), are heavily reliant on benchtop *ex vivo* microscopy. In this case, a technician must prepare and examine each individual sample slide to confirm the presence of a microorganism in a sample. The specificity and sensitivity of these tests is known to vary between 20 - 80% [5], with technician fatigue and experience impacting the outcome [5–7]. Indeed, sputum smear microscopy is the cornerstone of tuberculosis (TB) screening and identifies patients who may be infectious and a public health risk. Despite decades of progress in microscopy, smear microscopy remains a laboratory based process with slow turnaround and high resource requirements of trained staff [8]. The END TB initiative highlights the need for POC diagnostics and is key to reducing mortality [9].

Molecular-based fluorescent labels have the potential to lessen these requirements by increasing the specificity and sensitivity through increasing signal to noise ratios. However, it is not yet readily possible to use current benchtop fluorescence microscopes outside of the laboratory setting [10]. The microscope optics, both for white-light and fluorescence modalities, present a significant POC translational challenge. They often require expensive, specialized light sources, optics, and sensors and need additional power sources [10,11].

Developments have been made to address the need for lower cost, more accurate diagnostics in LMICs. Since the 2000s, complementary metal-oxide-semiconductor (CMOS) sensors have become an enabling technology due to their widespread employment in smartphones [12,13]. Today, CMOS sensors permit miniaturization, require less power, are inexpensive, and are readily available which has opened up their use as diagnostic devices at the POC [14–16]. Smartphones or other mobile devices, and single-board computers such as the raspberry Pi have combined sensors and communication tools including wireless internet (WiFi) and Bluetooth [17]. The advanced data compression functionality of CMOS sensors and the potential of mobile applications will allow *in situ* or remote diagnostics at an accurate level [18,19]. For LMICs, lower cost LED microscopy offers an alternative to expensive laser diode microscopes used in higher income countries [20].

In a fluorescence microscope an emission filter is used to block illumination light, allowing only the emitted fluorescence from the sample to be transmitted to the detector, increasing signal to noise ratio. Long pass filters are commonly used in fluorescence microscopy systems [21]. However, these components occupy valuable and limited space which can be a problem in a space-constrained handheld system where the lenses require a short focal length. Furthermore, the associated costs of multiple components can be prohibitively high [11]. In terms of sample preparation, recent chemical innovation has led to targeted ‘smart’ fluorescent stains that need little processing, acting as a switch when they come into contact with a bacterial cell membrane [22–24]. These labels may have major utility in settings where time and resources are limited such as in rural LMICs.

Cheaper optical components, including polydimethylsiloxane (PDMS) lenses have been developed and combined with smartphones [25,26]. PDMS has good optical characteristics such as transparency (T = 90 - 95%) [25,27] and is heat curable, meaning that it is possible to control its curing curvature, and thus the focal length of the lens. Previous work has indicated that for 10 µL heat cured PDMS lenses, a temperature of 200 °C gives rise to the greatest contact angle and magnification [25]. Additionally, this lens offers sufficient resolution for histological [25] and bacteriological imaging [21,28]. Smartphone microscopy systems have been developed with a resolution compatible with viruses and 100 nm nanoparticles, yet the use of laser diode light sources and expensive external lenses [29] may make cost a prohibitive factor for translation to a low resource setting. Low cost 3D printable structures including accurate stages could be used as a framework for any future imaging platform [30].

Here, we describe how a PDMS integrated filtering lens (IFL) doped with a silicone dye is able to act as both an emission filter and a lens for use at the POC. It has suitable imaging characteristics for detection of fluorescently labelled *Mycobacteria smegmatis* (a rare human-pathogen but commonly used as a laboratory model for Mtb) [31,32]. The IFLs negate the need for two separate components in a POC device, which has the advantage of reducing the required physical space in any device. At a cost of less than 0.02 USD per lens, these IFLs could contribute towards low cost POC diagnostic solutions.

## 2. Material and methods

In order to fully characterize the IFLs, both the filtering properties and imaging properties of the lenses were tested, before being demonstrated in conjunction with a smartphone to image fluorescently labelled *M. smegmatis*. The following sections describe: the methodology for manufacturing the PDMS IFLs (Sec. 2.1); characterization of the imaging and filtering properties of PDMS lenses (Sec. 2.2); and the fluorescent *M. smegmatis* slide preparation (Sec. 2.3).

### 2.1. PDMS filtering lens manufacture

PDMS solution (Sylgard 184, Merck) was mixed in manufacturer’s guidelines 10:1 of polymer to curative agent. Additionally, green or red silicone dye (Silc Pig Green or Red, Bentley Advanced Materials) was added to 1%, 3%, 5%, 7% or 10% by weight to the polymer mix. This was then vacuum degassed to remove bubbles and ensure overall good imaging quality through the lens. 10 µL or 25 µL of polymer was syringed onto a clean glass slide on a heat plate at a range of temperatures from 150 °C to 225 °C.

### 2.2. Filtering and imaging characterization of PDMS filtering lenses

#### 2.2.1. Setup for transmission measurement

The filtering effect of the lens with added dye was tested by placing the lens in the optical path of a white light source. The light source was collimated and the spot minimized using an iris before passing through the filtering lens which focused it into the spectrometer (USB2000+, Ocean Optics).

#### 2.2.2. Optical setup for lens focal length

To test the effect of the doping on the focal length of the lens, a 505 nm fibre coupled LED (M505F3, Thorlabs) setup was used. This incident light was collimated using a lens (C330TMD-A, Thorlabs) and minimized using an iris (SM1D12D, Thorlabs). A CMOS camera (DCC1645C, Thorlabs) was placed on a micrometer stage (XR50C/M, Thorlabs) so that the focal length of the lens could be calculated by finding the smallest spot size on the camera.

#### 2.2.3. Measurement of filtering lens contact angle

To measure the contact angle of each lens, a OnePlus 5 T smartphone (android 9) camera (16MP) was positioned fixed to the normal of the glass slide in a slide holder (XYF1/M, Thorlabs). The filtering lens was on the superior surface of the glass slide. Images were taken at maximum zoom (x8). Image analysis was performed using Fiji_64 Image J contact angle plug-in.

#### 2.2.4. Optical setup for resolution imaging

The imaging capabilities were tested using a white LED light source to illuminate a positive 1951 USAF resolution target for non-fluorescence imaging (R1DS1P, Thorlabs). The target was placed at the focal length of the filtering lens which was placed on the front camera of the OnePlus 5 T smartphone.

### 2.3. Fluorescent mycobacterial slide preparation

*M. smegmatis* (ATCC 23032) colonies were grown on 7H10 Middlebrook agar (Merck, catalogue M0303) supplemented with 10% v/v oleate-albumin-dextrose-catalase (OADC) (Merck cat. M0678), 0.5% glycerol (Merck, catalogue G5516) and sodium pyruvate (Merck, catalogue 5280) at 37 °C and stored at 4 °C. A single colony was smeared directly into an ‘all microbe’, NBD-based SmartProbe (30 µL, 5 µM) [22] on a clean glass microscope slide and mounted with a coverslip. Images were captured using the smartphone-IFL device setup as described above, or by wide-field microscopy (EVOS FL Imaging System, Thermo Scientific AMF4300) with GFP LED filter cube and 20x objective. Images were brightness and contrast enhanced using Fiji_64 Image J.

## 3. Results and discussion

### 3.1. Characterization of the filtering lenses

**Fig. 1.**
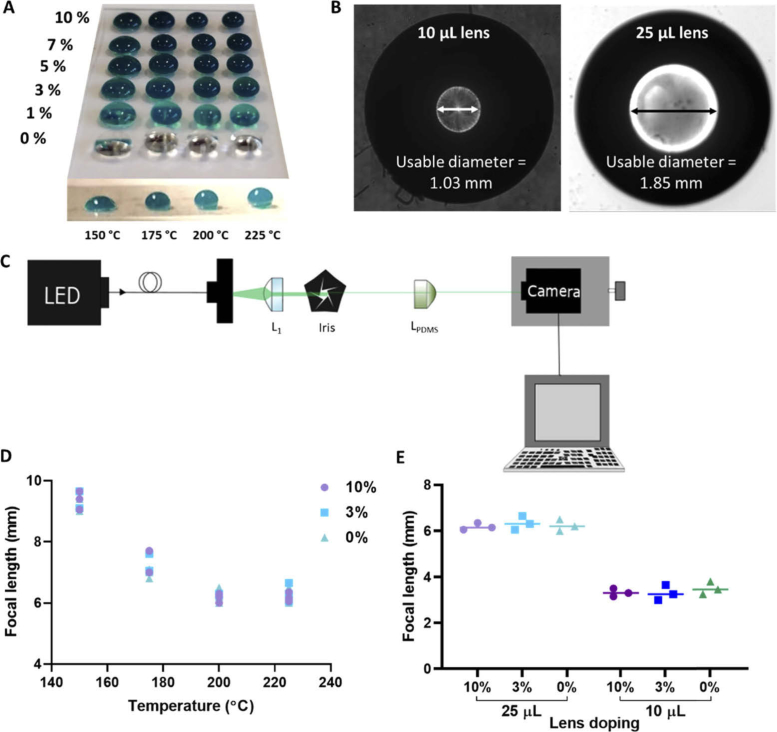
The doping in the PDMS lenses does not affect the ability for the lens to focus light. (A) Hand syringed filtering 25 µL PDMS lenses doped with green silicone pig dye at 0%, 1%, 3%, 5%, 7% and 10% by weight as shown in each row. The lenses were fabricated at increasing temperatures as shown in each column with 150 °C on the left, increasing to 225 °C on the right. (B) The usable diameter/clear aperture of the 10 µL and 25 µL lenses is 1.03 mm and 1.85 mm, respectively, as measured using Image J. (C) Focal length schematic showing LED light source attached to optical fibre with a collimating lens (L_1_), iris and PDMS lens (L_PDMS_). The camera is mounted onto a z-stage and attached to the computer. (D) With increasing temperature the focal length of the lenses reduced. The doping has no effect on the behavior of the lens with the 0%, 3% and 10% behaving with a similar pattern. Data shown for 25 µL lenses, with each lens shown by a single point. (E) The lower volume lenses (10 µL) have a smaller focal length than the larger 25 µL lenses, with the doping having little effect on the behavior of the lens. Each point represents a single filtering lens manufactured at 225 °C.

To investigate the effect of adding in a silicone pigment to the clear PDMS lens, fabrication for the 25 µL lenses occurred over a temperature range from 150 °C to 225 °C, and across a range of doping from 0% to 10% as shown in Fig. 1(A). Additionally, 10 µL lenses were fabricated at 225 °C. The average usable diameter, or clear aperture, was calculated using the lens on a CMOS detector. As expected, the larger (25 µL) lens has a greater clear aperture which gives a larger field-of-view (FOV).

Previous work from Sung *et al*. [25] demonstrated that the focal length, *f*, of clear PDMS lenses reduces with increasing temperature. Here, we observed that at temperatures of > 200 °C there was a plateauing of the focal length suggesting that for the 25 µL lenses there was a minimal achievable focal length (Fig. 1(D)). To compare the effect of volume on focal length, the 10 µL lenses fabricated at 225 °C were compared to the 25 µL lenses fabricated at the same temperature. The focal length of the 10 µL lenses was approximately half the focal length of the 25 µL lenses (Fig. 1(E)).

By comparing the 0% (i.e. clear) doped lenses, with 3% and 10% lenses it was found that the dye had no effect on the focal length of the lenses shown in Fig. 1(D), with there being no significant differee between any of the doping concentrations.

**Fig. 2.**
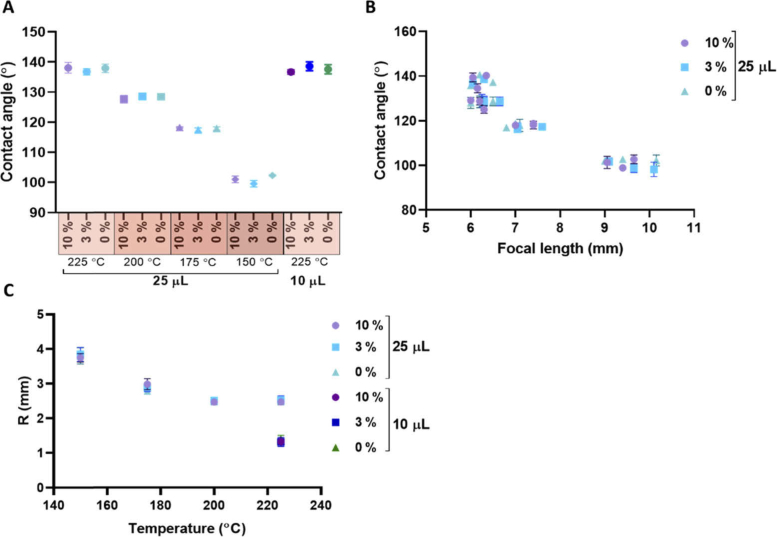
Adding a doping dye to the PDMS lenses does not alter geometry of the lens. The contact angles relative curvature were measured using the Image J plug-ins. (A) With increasing temperature the contact angle increased for the 25 µL lenses. The doping has no effect on the contact angle on the lens with the lenses within each temperature grouping having similar contact angle. Each point represents the average contact angle of three separate lenses, manufactured at a given temperature and doping, with error bars showing the s.e.m. (B) The contact angle correlates with the focal length, with greater variation at lower focal lengths. Doping the lens does not affect this trend. Each point represents a single lens with n = 3 IFLs measured for each temperature. (C) The relative curvature (R) of the lenses (proportional to the focal length) is dependent on temperature. The 10 µL lenses have a lower R. The doping has no effect on R. Each point is represented by the average of three lenses with error bars denoting the s.e.m of the group.

The focal length and temperature correlate as a result of the variation in the contact angle created at these temperatures. The PDMS cures more quickly at higher temperature and so gravity has less opportunity to take effect, resulting in a greater contact angle. The doping percentage of the PDMS lenses did not have an effect on the contact angle, shown in Fig. 2(A). There was no significant difference in contact angles between the 10 µL and the 25 µL lenses fabricated at 225 °C suggesting that at this temperature contact angle is independent of volume. Figure 2(B) shows correlation between contact angle and focal length. At a focal length of 6 mm, there is a greater range of contact angles than for higher focal lengths such as 10 mm.

The focal length of a lens is proportional to its radii of curvature and is expressed by the lensmaker’s equation. Taking the thin lens approximation,

(1) $$\frac{1}{f}\approx \frac{n_{2}-n_{1}}{n_{1}}\left(\frac{1}{R_{1}}-\frac{1}{R_{2}}\right),$$

where n_1_ and n_2_ are the refractive indices of air and PDMS, respectively, and R_1_ and R_2_ are the radii of curvature of the two surfaces. In the case of planar convex lenses, such as the PDMS IFLs, the term 1/R_1_ vanishes and the formula can be rearranged to express R_2_ as,

(2) $$R_{2}\approx 0.4f,$$

where we have taken the refractive index of PDMS to be 1.4. R_2_ decreases with temperatures from 150 °C to 200 °C and then plateaus from 200 °C to 225 °C shown in Fig. 2. The 10 µL lenses have a smaller R_2_ than the 25 µL lenses.

Figure 2(C) shows correlation between temperature and relative curvature (R) of the lenses. At the highest temperatures, there is greater variation in R, causing variation in focal length (observed in Fig. 2(B)), even though contact angle is repeatable.

It may be that contact angles are approaching a maximum achievable at high temperatures while R is still varying, however further work would need to investigate higher temperatures to clarify this. The contact angle will also be influenced by the drop height. There may have been an error introduced in fabrication with drop height only controlled to the nearest 5 mm. Additionally, the heat plate may not be of uniform heat across the surface and so the positioning of the glass slide during manufacture may have resulted in a slight spatial variation in temperature. This could have contributed to a slight variation in diameter/contact angle at each volume/temperature combination. Damodroa [27] found a similar effect to that observed here, with both *f*-number and NA being independent of temperature above a certain temperature (for 3 µL lenses this was 98 °C).

The doping is considered to have no effect on the curvature of the lens. This is important as the curvature of the lens will affect the NA. The NA of 0.27 is based on the usable radius of 1.03 mm and 1.86 mm for the 10 µL and 25 µL lenses, respectively. This is in keeping with previous work [25,27].

### 3.2. The PDMS integrated filtering lenses transmit in a concentration dependent manner

The purpose of doping the lens is to enable one component to act as both an emission filter and a lens. The filtering properties of the IFL need to behave in two ways; i) to block the incident light (excitation wavelength in the fluorescence system) and ii) to transmit the emitted light.

**Fig. 3.**
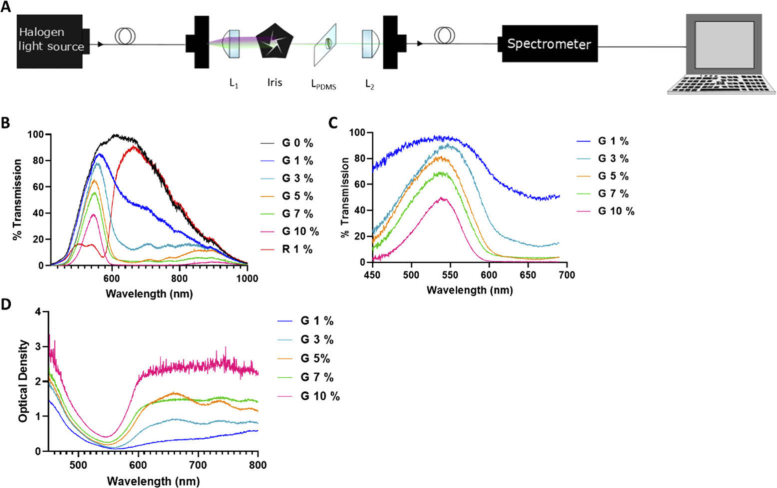
The IFLs have the ability to filter light in the visible spectrum. Transmission schematic (A) for setup with fibre coupled white light source into a collimating lens (L_1_), iris and PDMS filtering lens (L_PDMS_). There is an additional lens to focus the light into a second optical fibre (L_2_) feeding to the fibre coupled spectrometer. (B) % transmission spectra measured by the spectrometer for the green IFLs (labelled G) and red 1% IFLs (labelled R) over the visible spectrum. (C) Transmission spectra for green IFLs 1% to 10%. The green IFLs show a concentration dependent response with 10% lens blocking more light than the 1% IFLs across the spectrum. Transmission peak at 545 nm displaying the IFLs ability to transmit green light. (D) Optical density plot for the filtering lens showing that the higher doped IFLs had a greater optical density than the lower doped IFLs.

When green IFLs are compared to red IFLs and a clear PDMS lens, the ability to transmit/absorb light based on a specific wavelength becomes clear, as shown in Fig. 3(B). The red IFLs do not transmit green wavelengths whereas the green IFLs do, and the clear lens is able to transmit light across the wider visible spectrum. The percentage of doping in the green IFLs effects the transmission of light across the green wavelengths of light. The lower doping (1% and 3%) enable a higher percentage of transmission than higher doping (7% and 10%), allowing more of the fluorescence light to reach the detector in a fluorescence system. Therefore the lower doped IFLs are better at this behaviour (described as behavior ii) above).

On the contrary, the higher doped IFLs filter out a higher percentage of light towards the blue end of the spectrum. Commonly, fluorescence emission is red shifted away from the excitation wavelength, which means that the higher doped IFLs have a better ability to block the incident light (described as behavior i) above). However, the higher doped IFLs also act at a greater optical density across the full visible spectrum as shown in Fig. 3(D). The lower doped IFLs allow for a greater amount of the emitted wavelength light to pass through.

The lenses that were more effective in blocking the incident light were less effective at transmitting shifted light and vice versa. This means that a balance between these characteristics needs to be sought.

**Fig. 4.**
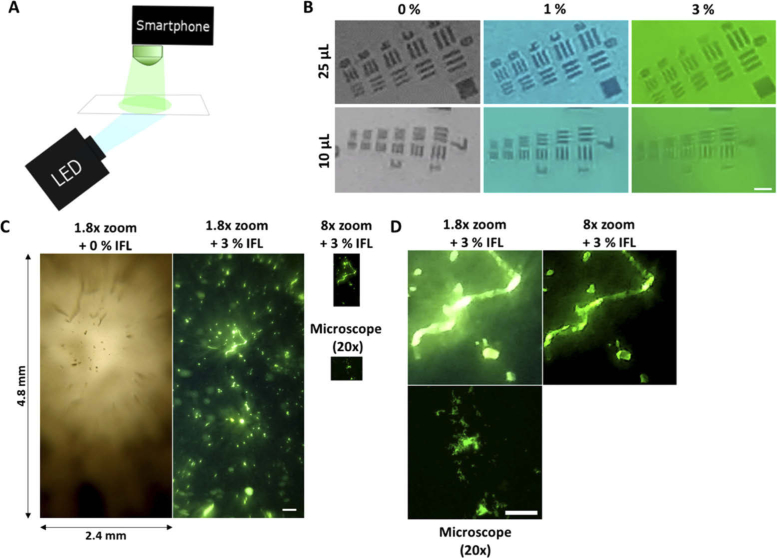
The filtering PDMS IFLs are able to image fluorescent targets with a resolution of 3 µm, including labelled *M. smegmatis* on a OnePlus 5 T smartphone. (A) Schematic of the smartphone microscope setup with IFL attached directly to the camera lens, with a glass slide bearing fluorescently labelled sample illuminated by a white LED light source. (B) Smartphone with PDMS IFL on the front camera imaging USAF targets. Top, left-right: positive USAF 1951 target Group 6 imaged with 25 µL IFLs at 0%, 1% and 3%. Resolution achieved is 4.4 µm. Bottom, left-right: positive USAF 1951 target Group 6 imaged with 10 µL IFLs at 0%, 1% and 3% IFL. Resolution achieved is 3.1 µm, scale bar shows 5 µm. (C) Representative imaging of fluorescently labelled *M. smegmatis* captured with the smartphone with either 0% or 3% doped IFL (digital zoom 1.8x or 8x) or wide-field fluorescent microscope (20x objective). All images shown to scale with full FOV captured. Scale bar shows 250 µm. (D) Fluorescent images shown in (C) scaled to show the same size FOV for comparison of image quality taken from the same location at 1.8 x zoom (left) and 8x zoom (right). Scale bar shows 100 µm.

The PDMS IFLs are appropriate for use with a fluorophore that emits in the green region of the visible spectrum above 520 nm and is excited by wavelengths < 470 nm, with the 10% IFLs giving the highest potential excitation blocking to emission transmission ratio. However, imaging with the various IFLs concluded that 3% doping balances the filtering effects with the light source power requirement, as shown in Fig. 4.

### 3.3. Imaging with the PDMS integrated filtering lenses

The PDMS IFLs were combined with a OnePlus 5 T smartphone running android 9, as shown in Fig. 4(A), to build an imaging system that could have an application at the POC. The specifications for the camera used for imaging were 16 MP and f = 20 mm, sensor 1/3.1 with a pixel size of 1 µm. The PDMS IFLs are self-adhesive to the camera on the phone.

Whilst Mtb characteristically appears as clumps of cells during sputum-smear microscopy, the average size of a single Mtb is 2 - 4 µM, therefore it is desirable for frugal imaging systems to have a resolution within this region. Using the 1951 USAF target (R1DS1P, Thorlabs) it was possible to determine the resolution of the 25 µL lens system to be less than 4 µm as shown in Fig. 4(B). The 10 µL IFLs have a greater resolution, at 3.1 µm, but the IFL is smaller than the smartphone camera and physical application of the IFL is more challenging than with the 25 µL IFLs. As discussed above, the 25 µL IFLs give a greater FOV which is beneficial when imaging over a greater area. These images were obtained using a white LED. Further work should investigate the most appropriate light source, including a blue LED with a fluorescent target.

The doping of the lenses impacted the required LED power to form an image. At a concentration of 1% or 3% dopant there was a sufficient filtering effect to remove light outside of the excitation spectrum, whilst allowing an image to form. Above this, the optical density was too great as it fully removed the required excitation light. Whilst we were able to obtain these high resolution images of the USAF target, we do observe noise within the images; these are jpeg compression artefacts; they are most prevalent at the high digital zoom levels which are required to display these images, and limits optical resolution.

Fluorescently labelled *M. smegmatis* was imaged with clear and 3% doped lenses by our IFL-smartphone microscopy platform. The *M. smegmatis* was labelled directly on the slide with a wash-free fluorescent SmartProbe, which enables the labelling of microbes within seconds. The images were compared with benchtop wide-field microscopy images of the same slides collected at 20x magnification. Figure 4(C) demonstrates the functional utility of developing doped lenses, despite imaging the same specimen with the same set-up no fluorescently labelled *M. smegmatis* are visible when using the clear lenses, that is compared to very distinct fluorescent puncta visible when the 3% IFL is used within the set-up. Moreover the field-of-view is on the order of 1 mm^2^ meaning that the operator would need to scan fewer fields compared to conventional fluorescence microscopy, and potentially false-negatives resultant from diffuse samples, or samples with low bacterial load (as is often the case for sputum smear microscopy of Mtb) could be reduced. Whilst we were able to achieve a much larger field-of-view with our smartphone setup at both 1.8x and 8x camera zoom compared to conventional wide-field fluorescence microscopy, we did not suffer from significant loss in resolution from our device. Figure 4(D) shows the same size field-of-view for each imaging set-up, and clumps of *M. smegmatis* of various sizes are easily distinguishable. Due to the low pathogenicity, but similar cell-wall structure and morphology of *M. smegmatis* to *M. tuberculosis* it is widely accepted as an *in vitro* model for the latter [33], thus it was selected as the model organism within this study, and we anticipate that similar fluorescence intensity and resolution could be achieved in subsequent TB imaging studies, however this warrants further biological evaluation.

## 4. Conclusion

We demonstrated here a potential application towards fluorescently labelled TB detection, which requires only the confirmation of the presence of the bacterial species, not benchtop microscope resolution of the microbe. As such, a TB screening IFL device could be realized with only a phone and a green dye doped IFL. The PDMS IFLs described here were able to combine two optical components, a lens and a filter, into a single low-cost solution that could be used at the POC. These lenses have a simple manufacturing protocol that can be easily scaled up for a cost of < 0.02 USD pp, with mass production ensuring control over contact angle and hence the focal length. This would ensure that smartphones with different camera specifications can be catered for with minimal IFL modification. In addition, a large number of fluorescent dyes exist, each with a variety of optical properties which could be leveraged to produce IFLs suitable for an array of diagnostic applications; and although beyond the scope of this work, 3D printed microscope stages are available open source and could add to the utility of a complete IFL-enabled smartphone microscope platform. In this way, we envision that the IFLs presented here will be suitable for a number of POC diagnostic applications, with the IFLs themselves being easily transportable and robust, adhering to ASSURED criteria [34].

The IFLs were combined with smartphone cameras, and whilst these require some energy, solar powered chargers are widely available or can be charged from a generator. Furthermore, applying the IFLs requires little training for non-skilled technicians to obtain an image, meaning a health care worker in a rural village could use them at the POC, store the data and then connect to hospitals or clinics with the results remotely. Whilst the pigment doping will only be compatible with certain fluorophores, this will be of advantage in a system designed to only detect one specific label, such as a specific stain for TB.

We focused here on green IFLs because the majority of fluorescent labels are in the green spectral range, like the nitrobenzoxadiazole (NBD) conjugated stain used in the imaging above. Although lenses with a variety of spectral filtering behaviours have been recently developed, the application here is specific for the POC [26]. In choosing the correct doping, a balance between the spectral filtering behaviours of blocking out incident light and transmitting emitted light is key, and the 3% doping was found to be adequate. Further work should investigate the red IFLs as this would increase the possible fluorescent labels that could be coupled into the system. The 25 µL IFLs offer a balance between FOV and resolution. They are easier to handle than the 10 µL lens and therefore maybe better suited to in-field applications.

One of the ongoing concerns in translating smartphone technology to the POC is the preparation of slides or biopsy. Currently there is limited advantage in progressing imaging capabilities while processing and fixation remains a barrier in the field. Future work into POC devices needs to address this concern if POC testing is to achieve its full potential in the future. Using ‘smart’, wash-free fluorescent labels, as demonstrated here, negates the need for multiple processing steps and may make this possible. Similarly, we captured all of the smartphone images using the camera’s automatic color balancing function, further demonstrating the ease of use of this imaging setup.

It is not purely a lack of technology that limits the implementation of POC devices in rural LMICs. In reality, the lack of consensus on procedure and definitions (despite national policy regarding contact tracing) continues to hamper progress [35] and so a multifaceted approach to engage leaders and experts will be needed for any POC device. Our vision is that the PDMS IFLs could be used in POC devices that use any smartphone with a camera app to enable in-field diagnostics. Such a device may have significant utility in settings such as POC sputum smear microscopy for TB, where accurately recording the presence or absence of the bacterium is paramount, particularly from diffuse samples or samples with low bacterial load. Evaluation of how our novel imaging platform performs on clinical sputum specimens from TB patients remains to be seen and offers an exciting continuation of study.

**DATA AVAILABILITY**: The experimental data is available via the Edinburgh DataShare (https://doi.org/10.7488/ds/2773).
